# The Effects of Physical Exercise with Music on Cognitive Function of Elderly People: Mihama-Kiho Project

**DOI:** 10.1371/journal.pone.0095230

**Published:** 2014-04-25

**Authors:** Masayuki Satoh, Jun-ichi Ogawa, Tomoko Tokita, Noriko Nakaguchi, Koji Nakao, Hirotaka Kida, Hidekazu Tomimoto

**Affiliations:** 1 Department of Dementia Prevention and Therapeutics, Graduate School of Medicine, Mie University, Tsu, Mie, Japan; 2 YAMAHA Music Foundation, Tokyo, Japan; 3 Department of Health and Welfare, Mihama Town Hall, Mihama, Mie, Japan; 4 Department of Health and Welfare, Kiho Town Hall, Kiho, Mie, Japan; 5 Department of Neurosurgery, Kinan Hospital, Tanabe, Japan; 6 Department of Neurology, Graduate School of Medicine, Mie University, Tsu, Mie, Japan; VU University Medical Center, Netherlands

## Abstract

**Background:**

Physical exercise has positive effects on cognitive function in elderly people. It is unknown, however, if combinations of non-pharmaceutical interventions can produce more benefits than single ones. This study aimed to identify if physical exercise combined with music improves cognitive function in normal elderly people more than exercise alone.

**Methods:**

We enrolled 119 subjects (age 65–84 years old). Forty subjects performed physical exercise (once a week for an hour with professional trainers) with musical accompaniment (ExM group), developed by YAMAHA Music Foundation; 40 subjects performed the same exercise without music (Ex group); 39 subjects were the control group (Cont group). Before and after the year-long intervention, each patient was assessed by neuropsychological batteries. MRIs were performed before and after intervention; the Voxel-based Specific Regional analysis system for Alzheimer's Disease (VSRAD) was used to assess medial temporal lobe atrophy.

**Results:**

Analysis of variance (ANOVA) was significant only in visuospatial function. The multiple comparison (ExM vs. Ex, ExM vs. Cont, Ex vs. Cont) was significant between the ExM and Cont group. Intra-group analyses before and after intervention revealed significant improvement in visuospatial function in the ExM group, and significant improvements in other batteries in all three groups. The VSRAD score significantly worsened in the ExM and Ex groups.

**Conclusions:**

Physical exercise combined with music produced more positive effects on cognitive function in elderly people than exercise alone. We attributed this improvement to the multifaceted nature of combining physical exercise with music, which can act simultaneously as both cognitive and physical training.

**Trial Registration:**

UMIN Clinical Trials Registry (UMIN-CTR) UMIN000012148

## Introduction

The positive effects of aerobic physical exercise on the prevention and occurrence of dementia have been well established. In the clinical practice guidelines for dementia edited by Societas Neurologica Japonica [1], aerobic exercise is given a recommendation grade B which means it is “Recommended to apply because of its scientific evidence”. The findings of the Honolulu Asia Aging Study suggested that walking was associated with a reduced risk of dementia [2]. Men who walked the least (<0.25 mile/day) experienced a 1.8-fold excess risk of dementia compared with those who walked more than 2 miles/day. Similar findings have been reported in many other studies [3]–[6]. In a randomized controlled trial of community-dwelling older adults, aerobic exercise training for 6 months increased hippocampal volume by 2%, effectively reversing age-related loss in volume by 1 to 2 years [7]. In contrast, hippocampal volume declined in the control group which only performed stretching exercise, although the volume change in the caudate nucleus and thalamus was insignificant between the aerobic exercise and control stretching group. These studies demonstrate that aerobic physical activity can have protective and regenerative effects against cognitive decline [8], and physical activity is also associated with improvement in attention [9] and executive function [10].

Other non-pharmaceutical interventions for the prevention of cognitive decline in dementia patients were ranked lower by the guidelines of the Societas Neurologica Japonica [1] which gave them only the of grade C1: “Recommended although there is no scientific evidence” [11]. Some reports have described the use of music therapy [12] in dementia patients for targeting cognitive dysfunction and behavioral and psychological symptoms of dementia (BPSD). By listening to music, patients with Alzheimer's disease (AD) showed improvement in categorical word fluency [13], autobiographical memory [14], and the memory of lyrics [15]. The efficacy of music therapy on BPSD has been demonstrated in recent systematic reviews [11], [16]. In healthy older adults, cognitive training interventions that offer practice in memory, attention, fine motor coordination, and visual and auditory processing have been associated with cognitive gains [8], [17], [18]. Parbery-Clark [19] reported that older musicians showed greater auditory working memory relative to non-musicians, and suggested that musical training might reduce the impact of age-related declines. Results from these studies suggest that music therapy might be useful for maintaining cognitive function in normal elderly adults and dementia patients.

It is reasonable to hypothesize that combining physical exercise with cognitive training will enhance cognitive function in normal elderly people more than can be achieved independently. To date, only three studies have reported on the effects of separate physical or cognitive activity training compared to the effects of combined training [8], [20], [21]. Fabre and his colleagues [20] compared aerobic training, mental training, and combined aerobic-mental training for two months among 32 healthy older adults aged 60–76 years old. Significant post-training improvements were observed in story memory, paired-associate learning, and memory quotient in the three trained groups. The mean difference in memory quotient pre- and post-training was significantly higher in the combined training group compared to either of the other two singular groups. The second study [21] evaluated the longitudinal effects of mental, physical, and combined training 1 year and 5 years after training, in a sample of 375 healthy, independently living older adults aged 75–93 years old. The extent of the positive effects in the cognitive training condition and in the combined condition were superior across a range of cognitive outcomes 1 year after training and were still evident 5 years later. Most recently, Shatil [8] studied whether combined cognitive training and physical activity training enhanced cognitive abilities more than either training regimen alone. Four groups of healthy older adults embarked on 4 months of cognitive and/or mild aerobic training: cognitive, mild aerobic training, a combination of both, and book-reading activity. The results indicated that older adults who engaged in cognitive training (separate or combined training groups) showed significant improvement in cognitive performance, compared to older adults who did not engage in cognitive training (the mild aerobic and control book-reading group). This suggests that the combination of physical exercise and cognitive training will improve cognitive function in healthy elderly people. As observed in the performance of figure skating and rhythmic gymnastics, music can facilitate and regulate physical exercise. Therefore, it is valid to assume that the combination of physical exercise with music will be superior to either alone, although such studies have yet to be conducted.

In the present study, we investigated the effect of physical exercise with and without music on cognitive function. Healthy elderly people participated in a physical exercise program once a week for one year. One group carried out the physical exercise with music accompaniment, and another without. Before and after the intervention period, neuropsychological assessment and brain MRI were carried out, and the results with and without musical accompaniment were compared.

## Subjects and Methods

### Subjects

**Figure 1:**
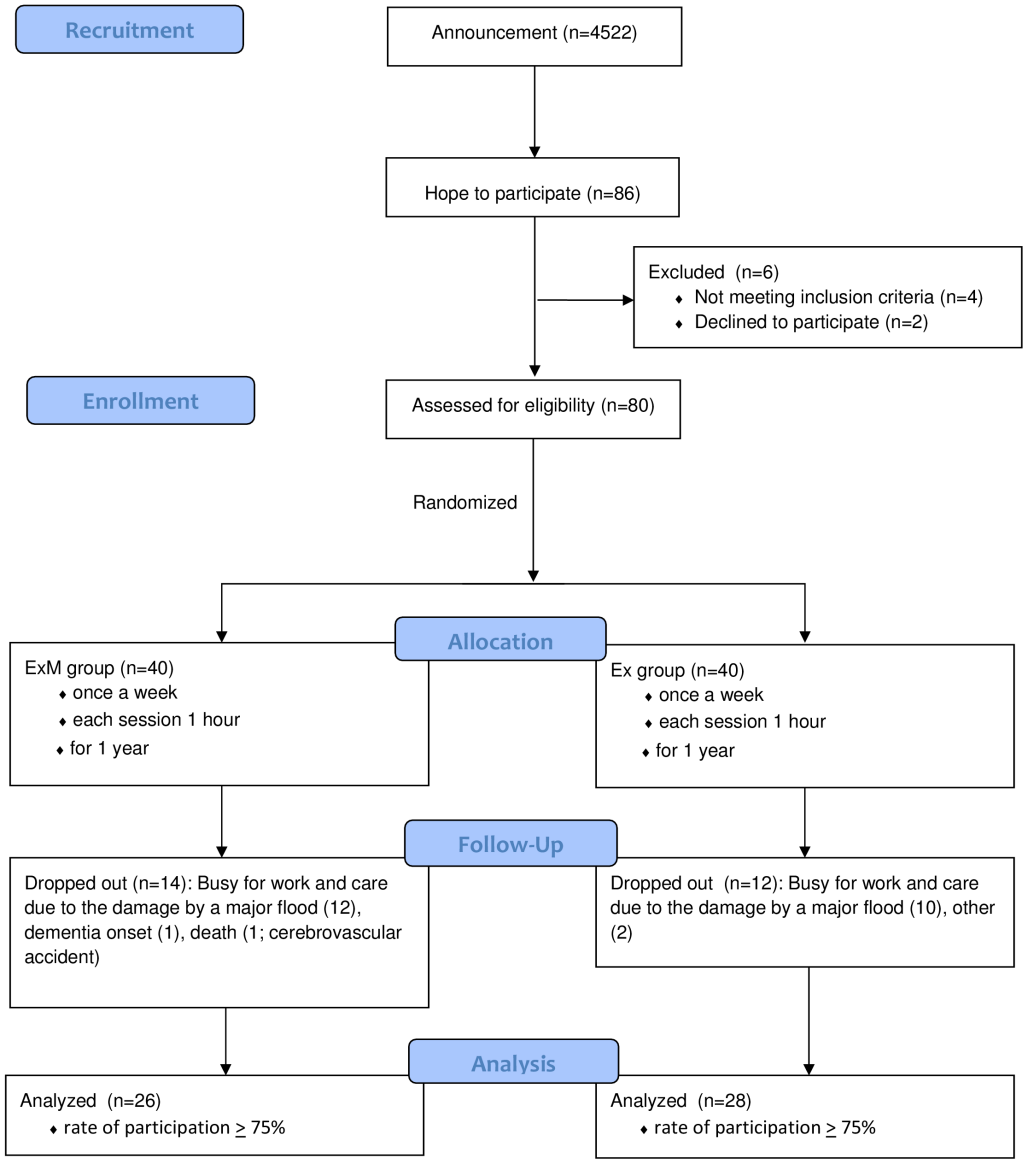
Flow Diagram of ExM and Ex group.

**Figure 2:**
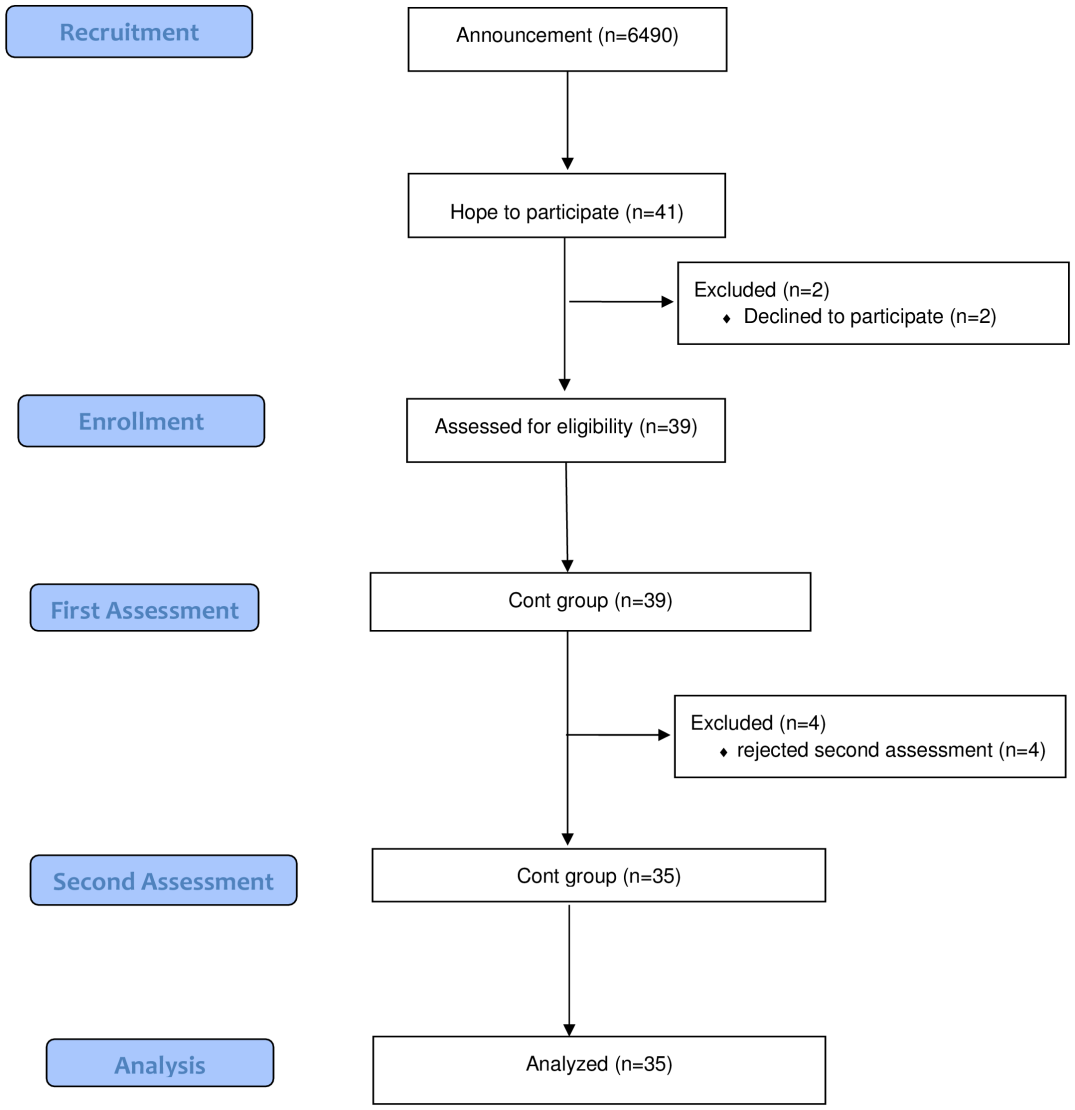
Flow Diagram of Cont group.

This study was conducted in the towns of Mihama and Kiho, which are situated at the southern end of the Kii peninsula in Japan. These towns suffer from depopulation and 35% of the population is over 65 years old (mean rate in Japan is 23%). It has been suggested that these towns represent the population that will exist throughout Japan in 20 years, therefore the prevention of dementia is an urgent problem of great importance. Based on the data of another city (Imari city, Saga prefecture, Japan) which had the almost the same populational structure as Mihama and Kiho town, we calculated the sample size as 123 (error 5, reliability 90, and population proportion 87%). This study was carried out as one of the public preventive projects for health of elderly people in these towns. The cost of this project was paid by the towns, and the available budget was limited to 120 subjects. We recruited participants via the distribution of paper fliers among the inhabitants of Mihama and Kiho town, which were sent by the public servants. The inclusion criteria were as follows: (a) over 65 years old, (b) in physically and psychologically healthy condition, (c) having corrected vision, (d) ability to clearly hear instructions, (e) living independently, and (f) able to be present once a week at the place of exercise. As for the physical and psychological condition, the public health nurses of each town saw participants and interviewed their family about physical and psychological activities of their daily lives. The participants were excluded if they met any of the following exclusion criteria: (a) apparent history of cerebrovascular attack, (b) the presence of chronic exhausting disease such as malignancy and infection, (c) the presence of severe cardiac, respiratory, and/or orthopedic disabilities which would prevent subjects from participating in exercise, (d) taking drugs that might adversely affect cognition (antidepressants and antipsychotics), and (e) having been diagnosed with dementia (Fig 1). We recruited 80 subjects from 1^st^ to 15^th^ of July, 2011, and, according to age, sex, and grade of the activity of daily life (ADL-grade) which was established by the Ministry of Health, Labour and Welfare (see Table S1), the subjects were semi-randomly classified into two groups: physical exercise with music group (ExM; age 65–83 years old; mean±sd 73.1±4.6; male 6, female 34; mean ADL-grade±sd 2.7±0.91) and exercise without music group (Ex; age 65–84 years old; mean±sd 73.3±4.8; male 5, female 35; mean ADL-grade±sd 2.7±0.98). The married couples, brothers and sisters who would like to come to the place of exercise were classified to the same group. The subjects of both groups participated in the exercise program for one hour once a week for one year. Before and after the intervention period, neuropsychological assessments and brain MRI were performed. As a control group (Cont), 39 subjects were also recruited during the same period of ExM and Ex group via paper fliers distributed among the inhabitants in Mihama and Kiho town (age 65–82 years old; mean±sd 73.5±5.6; male 8, female 31; mean ADL-grade±sd 2.8±1.1)(Fig 2). Because of the point of view of official services by town, the subject of Cont group were recruited apart from those of ExM and Ex group. They were examined two times with an interval of one year using the same neuropsychological batteries and imaging as the ExM and Ex groups. Using the age, educational history, ADL-grade, and the baseline score of MMSE as the independent variables, we carried out the crossed validation study between ExM, Ex, and Cont group, and all of the results were under 50%: ExM and Cont 48.3%, Ex and Cont 45.9%, and ExM and Ex 47.2%. We may say that the condition of these three groups were not so different. This study received approval from the Kinan Hospital Research Ethics Committee, and all patients provided written informed consent. This study was registered to UMIN-CTR (UMIN000012148) on 28^th^, October, 2013. Because this study was carried out as an official services by towns and approved by each town mayor, we did not think that the registration was needed, so the date of the registry was delayed. The authors confirm that all ongoing and related trials for this intervention are registered.

### Methods

The protocol for this trial and supporting TREND checklist are available as supporting information; see Checklist S1 and Protocol S1.

#### Physical Exercise

As an intervention, we performed physical exercise sessions for one hour with the ExM and Ex groups once a week. The intervention period was one year, and the total number of exercise sessions was 40. The exercise program was invented about 10 years ago under the collaboration of the Japan Fitness Association, experts of sport medicine, and the YAMAHA Music Foundation. The program consists of 9 stages (table 1), and, with appropriate musical accompaniment, participants perform the exercise easily and enjoyably. The exercise trainers were professional musicians who also held private licenses as physical trainers with the YAMAHA Music Foundation. The license is granted when referees of professional fitness examiners and sports medicine regard the applicant as being sufficiently skilled; the mean success rate is about 20%. The physical exercise regimen was identical for the ExM and Ex group. In the ExM group, however, music was played in harmony with the exercise, whereas the Ex group only heard a percussive sound which counted the beat. In stage 9, the singing portion, participants of the Ex group only read the lyrics aloud without the melody. The intensity of the exercise was gradually increased with each session.

**Table 1:** Contents of the exercise.

stage	title	duration (min.)	content
1	warming up	3	preparation
2	rhythmic walking	10	aerobic movement by marching
3	rhythmic exercise	5	clapping in various rhythms
4	muscle training	8	training of lumbar, limbs, and truncal muscles
5	stretch	5	stretching joints
6	respiration and voice	10	training of respiratory, facial, and speech muscles
7	rhythmic step	5	dancing
8	singing	10	singing familiar songs
9	cool down	2	relaxation

min.: minutes

#### Neuropsychological assessments

Before and after the one year intervention period, the following neuropsychological assessments were administered. To quantify intellectual function, the Mini-Mental State Examination (MMSE) and the Japanese Raven's Colored Progressive Matrices (RCPM) [22] were administered. RCPM measures not only the score but also the performance time which reflects the psychomotor speed of the subject. Memory was evaluated using logical memory I and II (LM-I/-II) of the Rivermead Behavioral Memory Test (RBMT) [23], which consisted of immediate and delayed recall of a short story. The RBMT has the four stories whose difficulties and the number of words and sentences are completely identical. We used the different story before and after the intervention period. The assessment of constructional ability was based on the method described by Strub & Black [24]. Five kinds of figures (vertical diamond, two-dimensional cross, three-dimensional block, three-dimensional pipe, and triangle within a triangle) were shown to the examinees and they were asked to draw them one by one. Each drawing was scored by assigning one of 4 possible grades (0: poor, 1: fair, 2: good, and 3: excellent), with a maximum score of 15. Frontal function was assessed by two types of tasks; word fluency (WF) and the Trail-Making Test A and B (TMT-A/-B). The WF test consisted of two domains: category and letters. For the categorical WF, subjects were asked to name as many animals as possible in one minute. For the WF of letters, for each of 4 phonemes *ka, sa, ta*, and *te*, the subjects were asked to say the name of objects which have that phoneme at the beginning of the word [25]. We used the average scores of these 4 phonemes for statistical analysis. It is generally accepted that the cognitive processing of the categorical and letter WF is different at least partially: the categorical WF reflects memory function more than the letter WF. These neuropsychological assessments were administered before and after the one-year music therapy intervention period to both the ExM and Ex groups. The subjects in the control group performed these assessments twice with an interval of one year.

Statistical analyses were performed as follows: first, analysis of variance (ANOVA) was carried out on the differences of each test before and after the intervention. If the result was significant, a multiple comparison (ExM vs. Ex, ExM vs. Cont, Ex vs. Cont) was done using the Mann-Whitney U test (Bonferroni correction, significance was defined as *p*<0.016). Second, for intra-group analyses, the results of each test before and after the intervention were compared in each group using the Wilcoxon signed rank test. All statistical analyses were performed by IBM SPSS Statistics software.

#### Voxel-based Specific Regional analysis system for Alzheimer's Disease (VSRAD)

The VSRAD was invented in 2003 [26]. It aims to show semi-quantitatively the degree of atrophy of the parahippocampal gyrus, for the purpose of diagnosing Alzheimer's disease (AD), and is based on statistical parametric mapping (SPM, Wellcome Trust Center for Imaging, London, UK; http://www.fil.ion.ucl.ac.uk/spm). Performing the VSRAD assessment was very easy and the analysis could be completed by a few clicks of a computer mouse. It is generally considered that if the VSRAD value is over 2, the patient might have AD, and if the value under 1, the subject might be normal. It is important to note that the VSRAD is just one biomarker of AD, so a diagnosis of AD should be based on the overall results from neurological, neuropsychological, biological, and neuroimaging findings.

## Results

During the one-year intervention period, sixteen subjects in the ExM group and twelve subjects in Ex group dropped out (Fig 1,2). Most of the reasons for dropping out were related to damages caused by a major flood which occurred in their towns in 2011 (the Kii peninsula great flood disaster). Four subjects in the Cont group rejected the second assessment after the one-year period. As a result, 26, 28, and 35 subjects of the ExM, Ex, and Cont groups were included in the analyses, respectively. Differences in age and baseline score on the MMSE were insignificant between subjects that had dropped out and those that completed the study in the ExM and Ex groups (age: ExM *p* = 0.26, Ex *p* = 0.40; MMSE: ExM *p* = 0.21, Ex *p* = 0.51). Differences in age, education, activities of daily life (ADL), and MMSE before the intervention in the ExM, Ex, and Cont groups were also insignificant (table 2).

**Table 2:** Characteristics of the ExM, Ex, and control groups.

	number (F:M)	age (±sd)	edu (±sd)	ADL (±sd)	MMSE (±sd)
ExM	26 (24∶2)	72.4 (3.7)	10.3 (1.6)	2.6 (0.9)	27.7 (2.3)
Ex	28 (24∶4)	72.8 (4.6)	10.8 (1.8)	2.6 (1.0)	27.9 (1.8)
Cont	35 (28∶7)	73.5 (5.5)	10.0 (2.1)	2.7 (1.1)	27.1 (2.6)

Kruskal-Wallis analysis (*p*-value)		0.75	0.68	0.88	0.41

ADL: activity of daily life, edu: education,

Ex: physical exercise without music, ExM: physical exercise with music.

F: female, M: male, MMSE: Mini-Mental State Examination.

sd: standard deviation.

### Neuropsychological assessments

The ANOVA showed significant results only in the visuospatial assessment (table 3, *p* = 0.006, Kruskal-Wallis test). The multiple comparison (ExM vs. Ex, ExM vs. Cont, Ex vs. Cont) was significant between the ExM and Cont groups (*p* = 0.002, Mann-Whitney U test), and, though insignificant, showed a trend towards improvement in the ExM compared to Ex group (*p* = 0.022, Mann-Whitney U test). The comparison between the Ex and Cont groups was insignificant (*p* = 0.421, Mann-Whitney U test).

**Table 3:** Results from the analysis of variance (ANOVA) of the extent of changes before and after intervention in the three groups.

test			difference before and after the intervention (mean(±sd))			*p*-value
			ExM	Ex	Cont	
**intelligence**	**MMSE**		1.0 (2.3)	−0.21 (2.1)	0.14 (2.6)	0.18^A^
	**RCPM**	score	0.6 (3.8)	−0.65 (3.1)	0.23 (3.5)	0.28^A^
		time	−44 (84)	−58 (94)	−19 (87)	0.16^B^
**memory**	**LM-I**		0.86 (3.3)	0.62 (3.7)	0.13 (4.1)	0.79^A^
	**LM-II**		1.4 (3.4)	1.1 (3.4)	1.4 (3.4)	0.96^A^
**visuospatial**	**copy**		1.7 (1.8)	0.57 (1.5)	0.26 (1.5)	**0.006** B
**frontal**	**WF**	animal	1.1 (4.4)	1.6 (4.5)	0.94 (5.6)	0.54^B^
		letters	0.6 (4.0)	0.0 (5.5)	0.0 (3.0)	0.63^B^
	**TMT**	-A	3.2 (29.9)	9.0 (34)	−8.1 (49)	0.54^B^
		-B	0.3 (46.1)	12 (69)	−37 (99)	0.086^B^
**MRI**	**VSRAD**		0.11 (0.18)	0.11 (0.27)	0.02 (0.28)	0.28^A^

A : 1 way ANOVA,

B : Kruskal-Wallis test, Cont: control group, Ex: exercise group, ExM: exercise with music group; LM: logical memory, MMSE: Mini-Mental State Examination, RCPM: Raven's Coloured Progressive Matrices, sd: standard deviation, TMT: Trail-Making Test, VSRAD: Voxel-based Specific Regional analysis system for Alzheimer's Disease; WF: word fluency; bold letters: significant.

The intra-group analyses (Wilcoxon signed rank test) before and after the intervention revealed some positive effects of the intervention (table 4). A significant improvement in visuospatial function was only observed in the ExM group (*p*<0.001). Other significant changes were as follows: MMSE (*p* = 0.041) and time of RCPM (*p* = 0.027) in the ExM group; time of RCPM (*p* = 0.002) and categorical WF (*p* = 0.026) in the Ex group; and LM-II (*p* = 0.036) and TMT-B (*p* = 0.008) in the Cont group. The VSRAD score significantly increased in the ExM (*p* = 0.007) and Ex groups (*p* = 0.030) suggesting a progression of degeneration in the medial temporal lobes.

**Table 4:** Results of intra-group analyses before and after the intervention.

tests				ExM		Ex		Cont	
				before	after	before	after	before	after
**intelligence**	**MMSE**		score (mean (sd))	27.7 (2.3)	28.7 (1.8)	27.9 (1.8)	27.8 (2.1)	27.1 (2.6)	27.2 (2.2)
			*p*-value	**0.041**		0.54		0.78	
	**RCPM**		score (mean (sd))	26.4 (5.6)	27.4 (5.2)	27.4 (3.3)	26.4 (4.1)	26.2 (4.6)	26.4 (3.8)
			*p*-value	0.29		0.28		0.70	
			time (seconds, mean (sd))	335 (98)	287 (62)	355 (115)	296 (91)	330 (134)	311 (104)
			*p*-value	**0.027**		**0.002**		0.26	
**memory**	**LM-I**		score (mean (sd))	11.3 (3.7)	12.1 (3.2)	11.6 (4.7)	12.1 (3.8)	10.5 (4.0)	10.6 (4.2)
			*p*-value	0.25		0.39		0.94	
	**LM-II**		score (mean (sd))	9.8 (4.4)	11.2 (3.6)	10.1 (4.1)	11.2 (4.2)	8.5 (3.9)	9.8 (4.6)
			*p*-value	0.075		0.10		**0.036**	
**visuospatial**	**copy**		score (mean (sd))	12.6 (1.6)	14.3 (1.3)	13.5 (1.3)	14.1 (1.2)	13.9 (1.3)	14.1 (1.2)
			*p*-value	**<0.001**		0.062		0.34	
**frontal**	**WF**	category	score (mean (sd))	14.7 (3.8)	15.7 (4.3)	12.9 (5.0)	14.7 (5.5)	12.4 (4.4)	13.4 (5.0)
			*p*-value	0.16		**0.026**		0.39	
		letters	score (mean (sd))	8.9 (3.0)	9.5 (3.3)	9.5 (2.8)	10.2 (5.3)	9.8 (4.6)	9.8 (3.7)
			*p*-value	0.33		0.88		0.93	
	**TMT**	-A	time (seconds, mean (sd))	127 (44)	130 (41)	137 (36)	145 (50)	140 (60)	133 (45)
			*p*-value	0.48		0.31		0.64	
		-B	time (seconds, mean (sd))	169 (61)	169 (45)	186 (77)	198 (85)	211 (123)	174 (63)
			*p*-value	0.81		0.41		**0.008**	
**MRI**	**VSRAD**		score (mean (sd))	0.84 (0.49)	0.95 (0.54)	0.91 (0.50)	1.02 (0.59)	1.32 (0.80)	1.34 (0.96)
			*p*-value	**0.007**		**0.030**		0.87	

Cont: control group, Ex: exercise group, ExM: exercise with music group, LM: logical memory, MMSE: Mini-Mental State Examination, RCPM: Raven's Coloured Progressive Matrices, sd: standard deviation, TMT: Trail-Making Test, VSRAD: Voxel-based Specific Regional analysis system for Alzheimer's Disease, WF: word fluency, bold letters: significant.

The results can be summarized as follows: i) the ExM group showed significant improvement in visuospatial function, ii) psychomotor speed might have been improved in the ExM and Ex groups, and iii) the ExM and Ex groups showed significant atrophy of the medial temporal lobes over the one-year period.

## Discussion

The present study suggests that music enhances the cognitive function of elderly people when combined with physical exercise. The content and duration of exercise were identical between the ExM and Ex groups, and the former showed significant improvement in visuospatial function compared to the Ex and Cont groups. The precise mechanism by which music facilitated the improvement is unknown, but data suggest three complimentary hypotheses. First, music might facilitate the efficacy of physical exercise itself. There are many cases in which music influences physical movement, for example marching, figure skating, rhythmic sportive gymnastics, and ballet. It is generally considered that appropriate musical accompaniment can positively influence movement, and vice versa. The content of the training program used in the present study was developed by a collaboration between professional gymnasts and musicians, so the high quality of the program might enhance the effects of the physical exercise. Second, physical exercise combined with music might act as both cognitive and physical training simultaneously, which would cause a greater improvement in the ExM group. Subjects in the ExM group listened to music, perceived the rhythm and the tempo of that music, judged whether their body movements and music were synchronous or not, and controlled their movements accordingly. The important factor is that these processes were performed simultaneously and continuously, such that the subjects were also receiving cognitive training during their physical exercise. The third hypothesis suggests that the parietal lobes might play a key role in the improvement that occurred with the musical accompaniment. It is well known that the parietal lobes participate in visuospatial and somatosensory function, including body image. During the physical exercise regimen, the subjects monitored the movement of their bodies and perceived the positions, postures, and acceleration of motion of their extremities. Several activation [27]–[32] and case studies [33], [34] have shown that the parietal lobes likely participate in music perception in the brain. Therefore, the stimulation of the parietal lobes by music and by the somatosensory inputs from physical exercise could cause improvements in visuospatial function.

The intra-group analyses showed significant improvement in the completion time of the RCPM in both the ExM and Ex groups. This finding suggests that physical exercise had a positive effect on psychomotor speed. Other significant changes were observed in the following neuropsychological assessments: the MMSE in the ExM group, categorical WF in the Ex group, and LM-II and TMT-B in the Cont group. One explanation for this variability may be attributed to individual differences in the subjects, though, as shown in Table 2, the general characteristics of the three groups were very similar. As described above, the improved MMSE in the ExM group might be caused by the combination of physical exercise with music by simultaneously acting as both cognitive and physical training. The categorical WF was significantly improved in Ex, but not in ExM group. The results of LM-II of both groups were improved after the intervention though statistically insignificant. We may say that the positive effect to memory by physical exercise might cause in the improvement of the categorical WF. Because the result of ExM group before the intervention period (14.7±3.8) was relatively better than that of Ex group (12.9±5.0), it is possible that the ‘ceiling effect’ might make the change of the categorical WF in ExM group insignificant. As for the Cont group, the baseline results of the LM-II and TMT-B were worse than those of the ExM and Ex groups. It is likely that the relatively poor baseline performance contributed to the observed changes in the Cont group after one year, though these results were within normal limits.

It must be noted that the values of the VSRAD had significantly worsened in the ExM and Ex group over the one-year period, indicating that in these groups, the atrophy of the medial temporal lobes including the parahippocampal gyrus had progressed. The values of these three groups were within normal limits, and the volume of parahippocampal gyrus of ExM and Ex group was relatively well preserved before the intervention. We may say that the aging effect caused in the significant changes of the VSRAD value in these groups, and the minimum change of the VSRAD value in Cont group might be the ceiling effect among cognitively normal elderly people. Some aspects of cognitive function were significantly improved, however, and no results indicated significant impairment of cognitive function within the year. It is possible that physical exercise not only compensates for the deterioration caused by normal aging and the progression of brain atrophy, but also shows benefits to cognitive function.

This study has several limitations. First, the number of the subjects was not very large. Further study with a larger number of participants might show other significant positive effects which, in this study, could not reach a level of significance, such as the time of the RCPM. Second, there might be a selection bias among subjects in the ExM and Ex group, because they were willing to attend the exercise program provided by their towns. Although the ADL-grade at baseline was the same between the three groups, it cannot be overlooked that the innate capacities of the subjects of the ExM and Ex groups might be different from those of the Cont group. Third, the intervention period was one year. The period of many other studies reported in the literature on the use of physical exercise and music therapy for the maintenance of cognitive function in dementia patients or normal elderly subjects was shorter than in the present study [7], [35]–[38]. A longer intervention period may produce more effects and prevent the progression of cognitive deterioration. Fourth, the attrition rates were not negligible. Because of the severe natural disaster occurred in 2011, some participants lost their houses, and the roads and bridges were destroyed. Some retired carpenters came back to their jobs for the restoration. They lost their time to participate in exercise classes. But, according to the public health nurses in the towns, the attrition rate in one year course of such projects to elderly people were usually over 50%. So, we may say that the attrition rate of the present study was not always so high. Lastly, it is impossible to completely deny the influence of the learning effect to the results of neuropsychological assessments. But, in the LM test, we used the different story before and after the intervention which had completely the same number of words, sentences, and difficulties. In the field of neuropsychology, there is fairly general agreement that the learning effect disappears after the interval of six months. The present results showed the significant difference between ExM, Ex, and Cont group which were assessed under the same interval. So, we may say that the learning effect would not contribute to the conclusion of the study. Further studies are needed to clarify the effects and mechanism of action of combining multiple intervention strategies for the improvement of cognitive function in elderly people.

### Summary

We performed physical exercise interventions with and without music in community-dwelling normal elderly people for one year, and neuropsychological changes in cognitive function were analyzed. The ExM group showed significant improvement in visuospatial function compared to the Ex and Cont groups. This effect might be attributable to the combination of two different strategies which act simultaneously as both cognitive and physical exercise training. We hypothesize that appropriate musical accompaniment can enhance the effects of physical exercise and help overcome the effects of brain atrophy caused by normal aging.

## Supporting Information

Table S1 Grades of activities of daily life from the Ministry of Health, Labour and Welfare in Japan.
(PDF)

Protocol S1 Trial Protocol.
(DOC)

Checklist S1 TREND Statement Checklist.
(PDF)
