# Correction: The Effects of Physical Exercise with Music on Cognitive Function of Elderly People: Mihama-Kiho Project

**DOI:** 10.1371/journal.pone.0111284

**Published:** 2014-10-10

**Authors:** The PLOS ONE Staff

There is an error in affiliation 5 for author Koji Nakao. Affiliation 5 should be: Department of Neurosurgery, Kinan Hospital, Mihama, Japan.
