# Supplementary material for: The Effects of Physical Exercise with Music on Cognitive Function of Elderly People: Mihama-Kiho Project
Source: PLoS One. 2014 Apr 25;9(4):e95230. doi: 10.1371/journal.pone.0095230 (PMC4000225; doi:10.1371/journal.pone.0095230)
Supplement: Table S1 — Grades of activities of daily life from the Ministry of Health, Labour and Welfare in Japan. (PDF) [file pone.0095230.s001.pdf]

Table S1. Grades of activities of daily life from the Ministry of Health, Labour and Welfare in Japan.

grade	content
I	Subject who has slight exercise each day. He/she takes a walk or shopping about an hour a day, and spends most of his/her time sitting.
II	Subject who has modarate exercise each day. He/she walks for two hours a day by commuting or working. At other times, he/she spends a lot of time sitting.
III	Subject who has strenuous exercise each day. He/she who fulfills grade II and engages in hard jobs such as agriculture and fishing for more than one hour each day.
IV	Subject who has very strenuous exercise each day. He/she spends more than one hour doing hard training, or engaged in forestry or agriculture in a busy farming season.
