# Supplementary material for: The Effects of Physical Exercise with Music on Cognitive Function of Elderly People: Mihama-Kiho Project
Source: PLoS One. 2014 Apr 25;9(4):e95230. doi: 10.1371/journal.pone.0095230 (PMC4000225; doi:10.1371/journal.pone.0095230)
Supplement: Protocol S1 — Trial Protocol. (DOC) [file pone.0095230.s002.doc]

**Dementia prevention for community-dwelling elderly people using physical exercise with music accompaniment: Mihama/Kiho Project**

**【protocol】**

**Persons concerned:**

Mihama Town Hall, Department of Health and Welfare: Tomoko Tokita

Kiho Town Hall, Department of Health and Welfare: Noriko Nakaguchi

YAMAHA Music Foundation, Department of Research and Development: Jun-ichi Ogawa, Yukio Kojima

Kinan Hospital, Neurosurgery: Koji Nakao

Community Cooperation Room: Hideshi Kanai

Mie University, Department of Dementia Prevention and Therapeutics: Masayuki Satoh, Hirotaka Kida, Hidekazu Tomimoto

**１．Name of the project**

Official: Mihama-Kiho Project

Popular name：”Mihama or Kiho Street Exercise”

1. **Systems of the towns**

In both Miahama and Kiho Town, this project is carried out as an official care prevention service for elderly residents.

1. **Subjects**

|  | **ExM group** | **Ex group** | **Cont group** | **total** |
| --- | --- | --- | --- | --- |
| Mihama Town | 20 | 20 | 20 | 60 |
| Kiho Town | 20 | 20 | 20 | 60 |
| total | 40 | 40 | 40 | 120 |

Ex: physical exercise without music, ExM: physical exercise with music, Cont: control

Inclusion criteria：(a) over 65 years old, (b) in physically and psychologically healthy condition, (c) having corrected vision, (d) ability to clearly hear instructions, (e) living independently, and (f) able to be present once a week at the place of exercise.

Exclusion criteria：(a) apparent history of cerebrovascular attack, (b) the presence of chronic exhausting disease such as malignancy and infection, (c) the presence of severe cardiac, respiratory, and/or orthopedic disabilities which would prevent subjects from participating in exercise, (d) taking drugs that might adversely affect cognition (antidepressants and antipsychotics), and (e) having been diagnosed with dementia.

Recruitment area：Miahama Town, Kiho Town

The way of recruitment：the distribution of paper fliers → holding an explanatory meeting in each town → acquisition of written informed consent

Grouping：We will recruit 80 subjects as the participants to exercise. According to age, sex, and grade of the activity of daily life established by the Ministry of Health, Labour and Welfare, the subjects are randomly classified into Ex and ExM groups. As a Cont group, 40 subjects are also recruited via paper fliers distributed among the inhabitants in Mihama and Kiho town. They are examined two times with an interval of one year using the same neuropsychological batteries and imaging as the ExM and Ex groups.

Place of the intervention：Friday

Mihama Town: “Kuroshio” Hall, in the morning.

　　　　　Kiho Town: Lesson Room in “Manabi no sato”, in the afternoon.

**４．Assessments (Brain Test)**

All following assessments are carried out in Kinan Hospital.

・Brain MRI ：T1, T2, Flair, sagittal T2, VSRAD, MRA

・Neuropsychological (below-mentioned)

・Blood：CBC、chemistry

・Physiological：ECG, respiratory

・Near Infra-Red Spectoroscopy (NIRS)：If it becomes possible.

Neuropsychological assessments

1. Mini-Mental State Examination (MMSE)
2. Immediate Memory: Logical memory-I, Rivermead Behavioral Memory Test (RBMT)
3. Raven’s Coloured Progressive Matrices (RCPM)
4. Trail Making Test-A/B (TMT-A/B)
5. Delayed memory: Logical memory-II, RBMT
6. Visuospatial ability
7. Word Fluency (animals, letters)

Report of results to participants

- The results are checked by Satoh, described in the sheet of “Report of Brain Test”, and send by mail to each participant from the town hall.
- In another day, an explanatory meeting of results of Brain Test will be held.
- If unexpected abnormality is found, the subject will be treated in Kinan Hospital.

**５．Schedule**

|  | **2011/July** | | **August** | **September** | | **October** | **November** | **December** | **2012/January** | **February** | **March** |
| --- | --- | --- | --- | --- | --- | --- | --- | --- | --- | --- | --- |
| **Mihama** | recruitment | Explanatory meeting | **Brain Test (pre-intervention)**  Kinan Hospital | |  | **Intervention (Mihama Town, “Kuroshio” Hall)**  Friday morning | | | | | |
| **Kiho** |  |  |  | | **Brain Test (pre-intervention)**  Kinan Hospital | | **Intervention (Kiho Town, lesson room of “Manabi no Sato”)**  Friday afternoon | | | | |
| **Cont** |  |  | | | | | **Brain Test**  Kinan Hospital | |  | | |

|  | **April** | **May** | **June** | **July** | **August** | **September** | **October** | **November** | **December** |
| --- | --- | --- | --- | --- | --- | --- | --- | --- | --- |
| **Mihama** | **Intervention (Mihama Town, “Kuroshio” Hall) (continued)**  Friday morning | | | | | | **Brain Test (post-intervention)**  Kinan Hospital |  | |
| **Kiho** | **Intervention (Kiho Town, lesson room of “Manabi no Sato”) (continued)**  Friday afternoon | | | | | | | **Brain Test (post-intervention)**  Kinan Hospital |  |
| **Cont** |  | | | | | | | | **Brain Test**  Kinan Hospital |
